# Development of Community-Level Capacity of Resilience to Natural Hazards for Environmental- and Social-Justice-Challenged Communities: 1. Approach, Concepts, and Assessment of Existing Information

**DOI:** 10.3390/su16030963

**Published:** 2024-01-23

**Authors:** J. Kevin Summers, Rachelle Sanderson, Rachelle Trahan, Kendra Hendricks, Mia Ruffin, Adam Williams, Andrea Lamper, Mason Lowery, Linda C. Harwell

**Affiliations:** 1 Gulf Ecosystem Measurement and Modeling Division, 1 Sabine Island Drive, Gulf Breeze, FL 32561, USA; 2 Capital Region Planning Commission, 14734 S. Harrell’s Ferry Road, Suite B, Baton Rouge, LA 70816, USA; 3 Institute for Sustainable Communities, Hammond, LA 70401, USA; 4 Coastal Sustainability Studio, Lafayette, LA 70503, USA; 5 Build Baton Rouge, 725 Main Street, Baton Rouge, LA 70802, USA; 6 Oak Ridge Associated Universities, 1 Sabine Island Drive, Gulf Breeze, FL 32561, USA; 7 CDM Smith, 670 N Commercial St., Unit 208, Manchester, NH 03101, USA; 8 Oasis Systems, LLC, 306 West F Avenue, Eglin AFB, FL 32542, USA

**Keywords:** resilience, community, capacity building, CRSI, climate, natural hazards

## Abstract

Impoverished and under-served communities are often exposed to the worst environmental and climate hazards. Identifying these communities and building their resilience capacity to withstand such hazards is a vital justice aspect of environmental management. Building community resilience requires five activities: (1) examination of existing information, (2) community engagement and assessment of local knowledge, (3) development of reasonable strategies to build resilience, (4) implementation and these strategies, and (5) monitoring and transability of the process. This manuscript examines the first component of this process. The attributes of multiple parishes in Louisiana are examined using available data and existing models of human well-being, community resilience, and environmental/climate/socioeconomic justice. These existing models and tools were used to determine parish-level resilience to natural hazards including flooding, hurricanes, and other potential natural climatic hazards in central Louisiana (U.S.). Through consultation with state officials and local community groups, candidate environmental justice (EJ) and social justice (SJ) communities were selected to develop resilience capacity enhancement plans to address potential adverse parish and community outcomes of natural hazard events. Of the available parishes, St. Helena Parish was selected as an entity that would significantly benefit from resilience capacity building. The remaining two activities, community engagement and strategy development, will be examined in sister manuscripts. Continuing studies, to be described elsewhere, will describe community engagement and the determination of strategies, implementation plans, and the monitoring of the success of these strategic implementations.

## 1. Introduction

Community resilience, as well as community resilience capacity building, are terms with many embedded transdisciplinary meanings. The literature on these topics spans five decades. Holling [1] initially introduced the term “resilience” from an ecological and systems perspective as a “measure of the persistence of systems and their ability to absorb change and disturbance and maintain the same relationships between populations or state variables.” Since then, numerous disciplines have adopted the term “resilience” in many contexts, including socioecological, urban, community, and disaster contexts [2]. Despite its often-contradictory meanings, resilience is a valuable bridge entry used to examine sustainability across community sectors and actors. In addressing community resilience, one must consider the many perspectives and stakeholders that make up the governance of a community [3].

Building resilience capacity in communities requires three elements—assessment of existing information, community engagement and evaluation of local knowledge, and development and implementation of strategies to enhance resilience capacity. This manuscript addresses the first element of this triad, and further manuscripts elsewhere will address each of the remaining elements.

The Southeastern Louisiana Resilience Capacity Building Project will assess one of the primary application areas of community resilience—natural hazard resilience; however, it will employ three approaches in its application—sustainability/equilibrium, adaptation, and transformation. Each approach operates efficiently within the application fields of quantification, spatial and temporal scales, community engagement, and equity. Resilience equilibrium focuses on a community’s ability to maintain services and functioning while absorbing a disaster’s impacts [4,5]. Community recovery is based on the ability to return to the previous equilibrium state or a new level of equilibrium with greater or lesser functionality [6–8]. A community’s capacity to bounce back represents an ability to resist disruption [9] by utilizing existing resources while ensuring business continuity, minimizing overall disruption, and preserving the built environment [10]. The concept of bouncing forward or building back better is the next step in the process, which targets the development of a new state of equilibrium that is better than the equilibrium that existed before the disruption [11]. Building back better provides a social opportunity to enhance recovery processes that help solve persistent community resilience inequalities and better meet the recovery needs of vulnerable or marginalized groups [12,13].

Adaptation occurs when a community changes in response to a disturbance [14]. An adaptive community is flexible and can incorporate new knowledge and technology to facilitate change after a disturbance [15]. Equilibrium and adaptation both tend toward reaching stability or some goal; transformation targets a complete change of the system or community to address vulnerabilities and inequalities without a visible endpoint [9,16–18].

Finally, the resilience framework must be applied equitably across all community areas. All communities have inherent resilience, but not all groups within a community have the same starting point to respond to disruption [19]. The concept of “bouncing back” relies on existing social, economic, or infrastructure capital [20] and reinforces challenges associated with equity [21].

The work presented here addresses the selection of target parishes and communities to establish community engagement activities. In this project, we sought to determine a subset of parishes in southeastern Louisiana (USA) (parishes are the civil government equivalent of counties in Louisiana) that can be characterized as low-resilience, low-well-being, equitability challenged for disaster recovery, and likely to benefit from resilience capacity building. After selecting the parishes through community engagement, we propose assessing local knowledge further to support the selections, utilizing these engagements to determine approaches and opportunities for resilience capacity building, implementing them, and assessing and monitoring their success.

## 2. Methods

**Figure 1.**
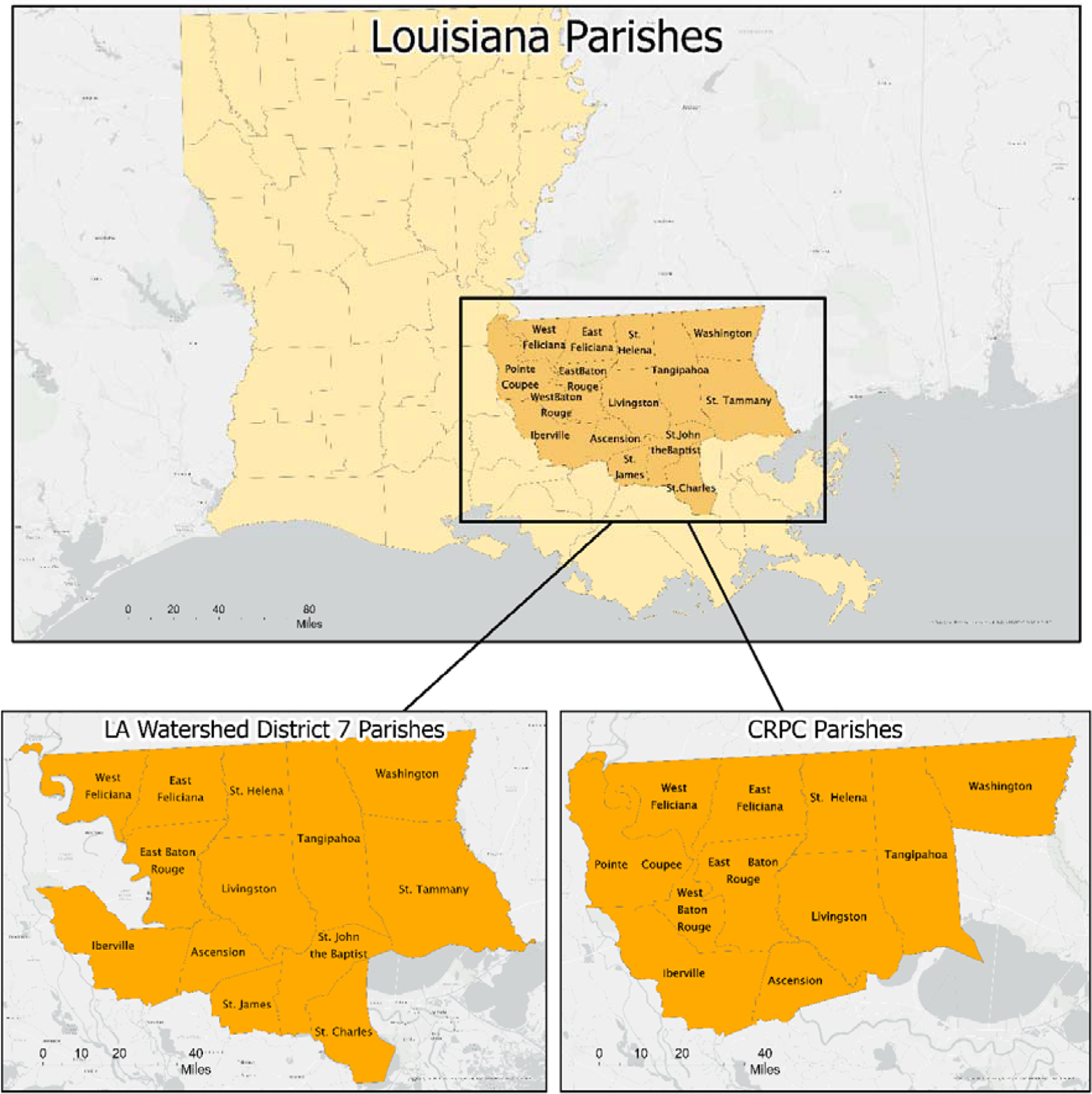
Targeted parishes from southeastern Louisiana representing the Capital Region Planning Commission and the Louisiana Watershed District 7.

The Capital Region Planning Commission (CRPC) serves eleven parishes in southeastern Louisiana and overlaps with many of the thirteen parishes encompassed by Louisiana Watershed District #7 (LAWD7) (https://watershed.la.gov/watershed-regions) (accessed on 12 November 23) (Figure 1). CRPC is the fiscal agent for the Louisiana Watershed District #7 unit. The fifteen parishes comprising CRPC and LAWD7 are rated as having poor resilience compared to other counties/parishes in the United States (mean CRSI score for U.S. = 3.06) based on the Cumulative Resilience Screening Index [22]. They are considered potential candidates for resilience capacity building.

**Figure 2.**
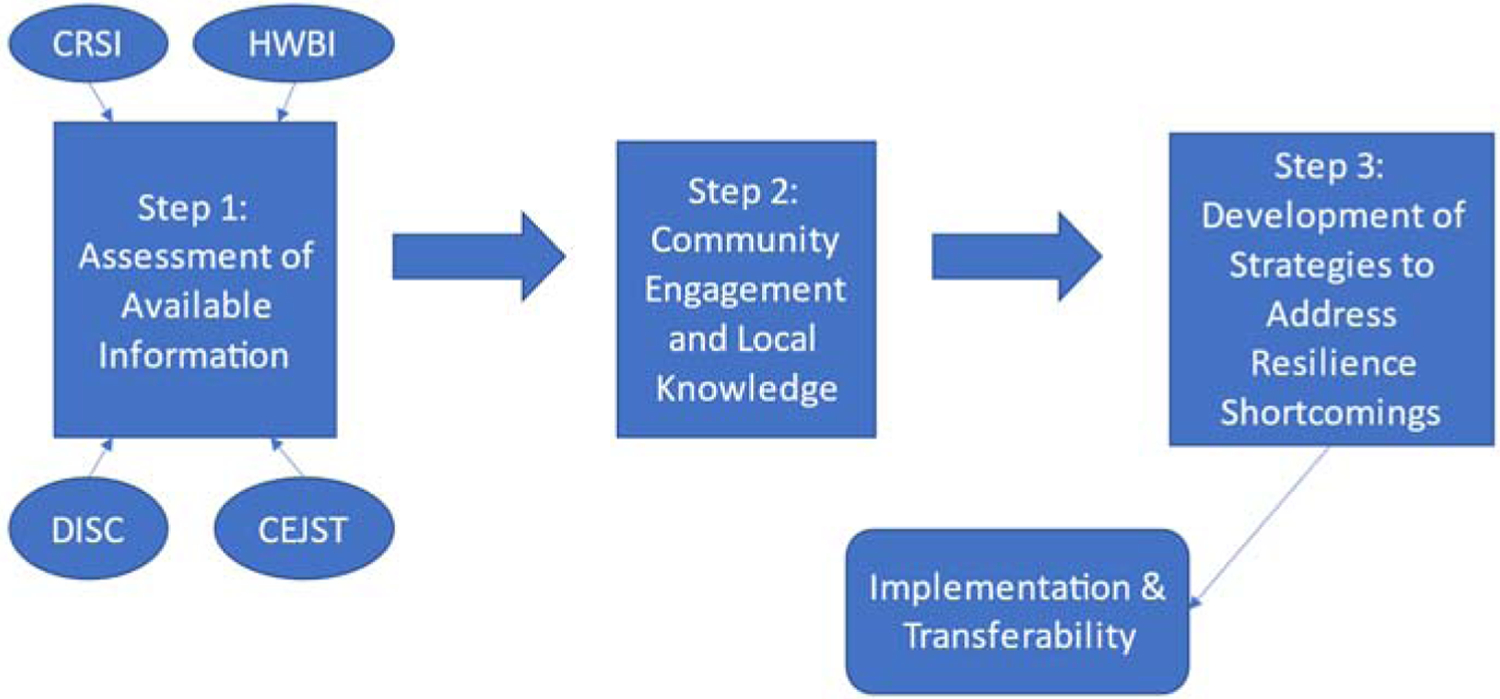
Conceptual model of resilience capacity building.

The conceptualization of this resilience capacity building process involves three major steps followed by implementation and assessment of transferability (Figure 2). The first step is examining the available data for the subject fifteen parishes. The examination uses available information to assess the well-being, the resilience to natural hazards, and the environmental/climate/socioeconomic attributes of each parish. The models used in this assessment are the Climate Resilience Screening Index [22,23], The Human Well-Being Index [24,25], the Decision Integration for Stronger Communities Tool (DISC) [26], the Environmental Justice Screening Tool (EJScreen) [27], and the Climate and Economic Justice Screening Tool (CEJST) [28]. Based on these model results and expert opinion, a parish is selected for resilience capacity building, The second step of the capacity building process is community engagement and the assessment of local knowledge to confirm the findings of the available data. The third and final step of the resilience building process is the development of strategies to address the resilience shortcomings identified in Steps 1 and 2. Steps 2 and 3 will be addressed in companion manuscripts. The assessment of available data (Step 1) is the subject of this paper. These three steps would be followed by local implementation of the strategies developed in Step 3 and the examination of the transferability of the three-step process to other Louisiana parishes and U.S. counties.

**Figure 3.**
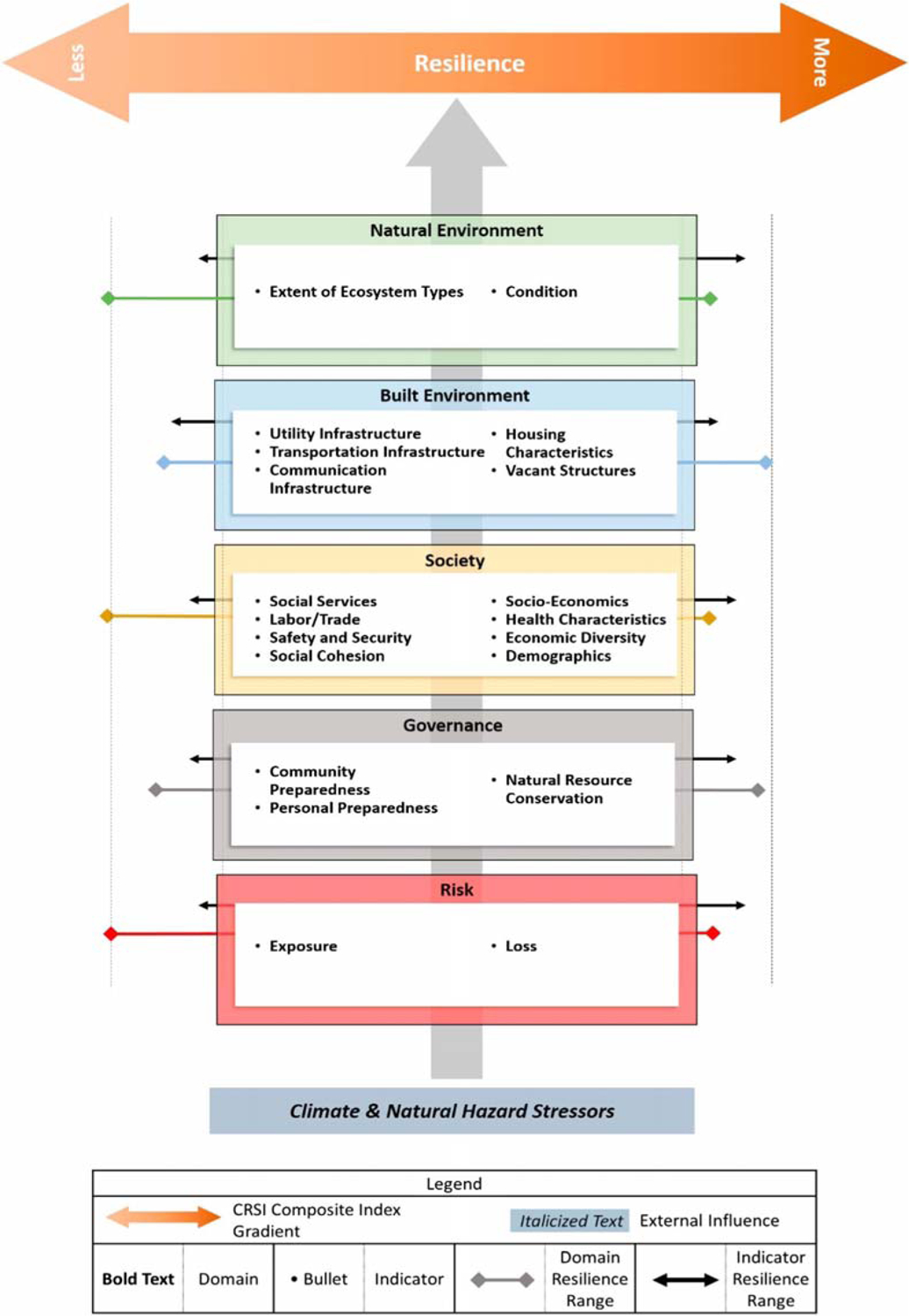
Conceptual framework for the Climate Resilience Screening Index (Summers et al. 2017, 2020) [22,23]. Domains are five large boxes, and indicators are bullet points in each box.

The conceptualization and structure of the Climate Resilience Screening Index (CRSI) is discussed fully by Summers et al. [22,23]. CRSI includes five domains (i.e., risk, governance, society, built environment, and natural environment) composed of 20 indicators (Figure 3), which, in turn, were derived from 117 unique metrics (see [23]). The metrics comprise data readily collected from existing surveys (e.g., American Community Survey), internet datasets (e.g., National Broadband Map Datasets), national and state statistics (e.g., National Bridge Inventory), and several other resources (e.g., NOAA Sea Level Rise Predictions, Facility Registry Service).

Following the concept of basic community resilience to natural hazards being driven by the likelihood of an event occurring and the community’s preparation for such an event, the domains of risk and governance are included at the base of the conceptual model (Figure 3) and develop a basic resilience score (i.e., the score of governance/score for risk). Exposure and losses comprise the indicators for risk, and community preparedness, personal preparedness, and natural resource conservation represent the governance indicators. Twenty metrics (e.g., exposure to hurricanes, high temperatures, loss of human life, loss of property) contribute to the two indicators (i.e., Exposure and Loss) of Risk, and five metrics (e.g., Average Community Rating System Score) contribute to the three indicators (i.e., Community Preparedness, Individual Preparedness, and Natural Resource Conservation) of Governance. The remaining domains include elements that could exacerbate or diminish a community’s vulnerability and recovery potential. These include society, the built environment, and the natural environment. Societal indicators include the availability of social services, available labor and trade services, safety and security requirements, socioeconomics and economic diversity, health characteristics and healthcare access, basic demographic information, and social cohesiveness. Fifty metrics are utilized to describe these societal indicators. The built-environment indicators include infrastructure elements—communications, utilities, and transportation—and housing characteristics. Twenty-four metrics are compiled to represent the built environment. The natural environment domain describes the resilience of natural and managed ecosystems through indicators of ecosystem extent and ecosystem condition. Eighteen metrics are combined to represent the indicators within the natural environment domain.

The fifteen parishes were characterized using the Cumulative Resilience Screening Index (CRSI) [22], the Human Well-Being Index (HWBI) [24,25], the Decision Integration for Stronger Communities Tool (DISC) [26], the Environmental Justice Screening Tool (EJScreen) [27], and the Climate and Economic Justice Screening Tool (CEJST) [28]. These scores were compared to determine which parishes could have low resilience to natural hazards, low well-being, low combined resilience and well-being, and high levels of inequity. The scores were also mapped using ARCGIS-Pro to provide visual comparisons. Finally, the fifteen parishes were assessed sociopolitically and practically to determine which parishes had the governance infrastructure necessary to undertake the project. These practical evaluations focused on the proximity of new elections and ease of working with and interest of specific parish officials. The fifteen parishes were reduced to four candidate parishes based on index and model scores in conjunction with the sociopolitical assessment.

The CRSI scores for the final four parishes, characterized by social and economic inequities, low resilience, and low well-being, were deconstructed to determine the primary indicators and metrics contributing to those high and low levels of resilience and well-being. These contributions were tabularly tallied and visually depicted using polar plots [22]. CRPC conducted a final evaluation to determine which among the four final candidate parishes would be selected for community engagement.

## 3. Results

The results of the indices for CRSI, HWBI, DISC, EJScreen, and CEJST as applied to the CRPC and Louisiana Watershed District #7 areas are shown in Table 1. The table provides the index scores for CRSI, HWBI, and DISC. The scores for all these indices theoretically range from 0 to 100. However, most county/parish scores for CRSI range from 0 to 10; for HWBI and DISC, most county/parish scores range from 30 to 70. For CRSI, very poor resilience is denoted by values below 1.0, and poor resilience by values of less than 2. For HWBI and DISC, very poor county/parish-level well-being is characterized by scores below 50, and poor well-being by scores below 55. For EJScreen calculations in Table 1, high environmental exposure to toxicants and other pollutants is characterized by scores above 60. Scores for EJScreen range from 0 to 100 and represent the mean score for the included indicators. For CEJST, parishes are considered very climate-challenged or socially challenged if greater than 50% of their census tracks are described as “disadvantaged”. Scores range for CEJST from 0% to 100%.

All of the parishes in the CRPC and Louisiana Watershed District #7 areas were below the mean CRSI scores for the combined counties of the United States (3.060) and below the combined parishes for the State of Louisiana (2.501). Three parishes displayed extremely poor resilience (<1.00)—Washington, Ascension, and St. Tammany parishes. Eight parishes displayed poor resilience (1.00 < × < 2.00)—East Feliciana, St. Helena, East Baton Rouge, Livingston, St. James, St. John the Baptist, Iberville, St. Charles, and West Baton Rouge parishes. All but three CRPC-represented parishes had poor to extremely poor CRSI scores (<2.5 mean for Louisiana parishes).

The mean scores for the parishes represented by the CRPC were among the lowest in the country. Roughly half of the CRPC and LW #7 parishes were below the mean Louisiana parish HWBI score of 49.0. All but two parishes (Ascension and St. Charles parishes) were below the mean United States county HWBI score of 54.1. Six parishes—East Feliciana, East Baton Rouge, Iberville, St. Helena, Tangipahoa, and Washington parishes—were in the bottom 3% of county HWBI scores in the United States. Similarly, these same parishes were in the bottom 5% of county DISC scores in the United States.

EJScreen identifies census tracts that experience high exposure to toxic waste in conjunction with skewed racial demographics (represented by heavily concentrated minority areas). All but two CRPC parishes (Ascension and Livingston Parishes) had more than 50% of their census tracts experiencing these conditions. In St. John the Baptist, East Baton Rouge, and Iberville parishes, with substantial shares of minority populations, over 73% of tracts exhibited high exposure to toxic substances. In East Feliciana, St. Helena, Tangipahoa, and Washington parishes, over 60% of the census tracts had high toxic exposures to their significant minority populations.

CEJST scores showed that greater than 60% of the census tracts in East Feliciana, Iberville, Tangipahoa, Washington, and St. John the Baptist parishes showed high climate and economic inequity levels. While only 50% of the census tracts in St. Helena Parish showed this level of inequity, the one inequitable tract (one of two in the parish) represented roughly two-thirds of its population. Approximately half of the CRPC parishes were exposed to toxic substances, experienced high climate justice inequity, and had poor socioeconomic conditions.

**Figure 4.**
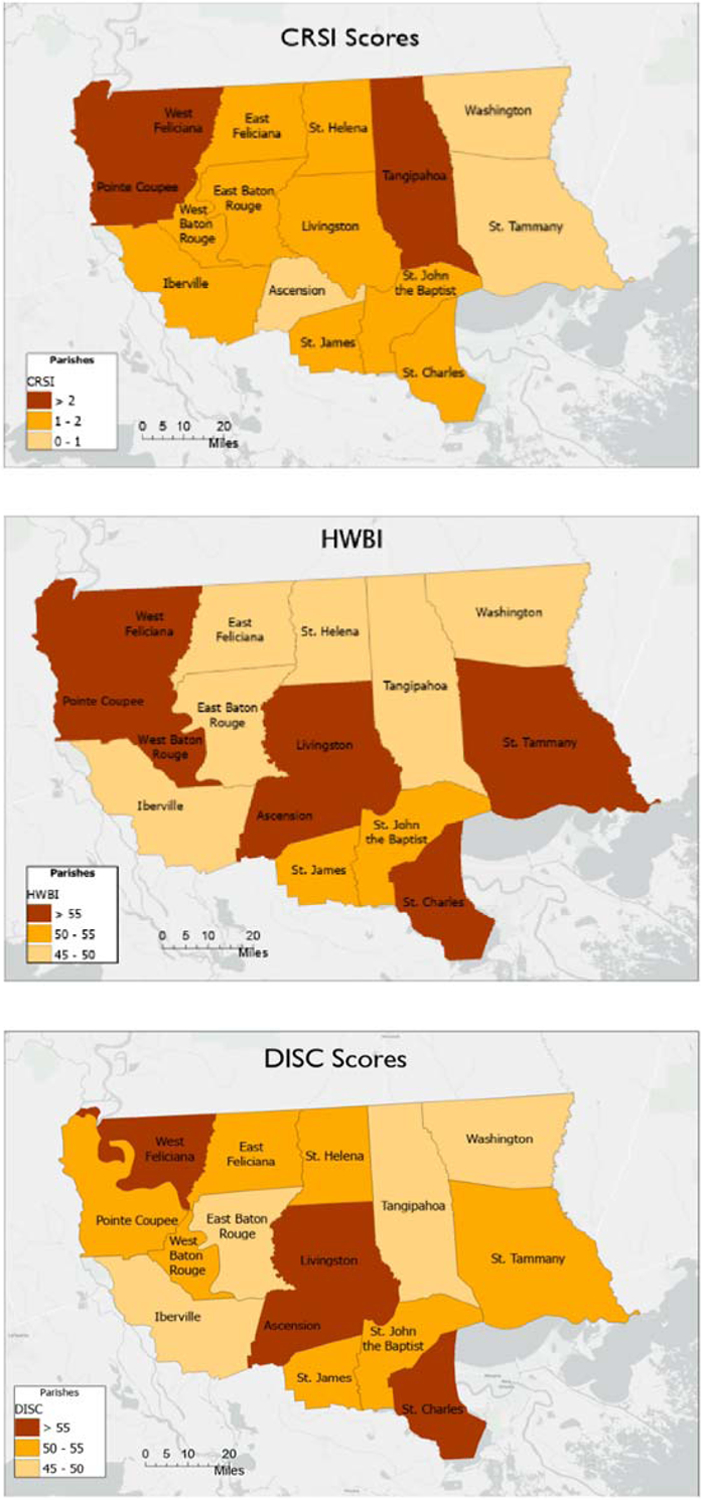
Spatial distribution of CRSI, HWBI, and DISC scores for targeted parishes.

The spatial distribution of CRSI, HWBI, and DISC scores is shown in Figure 4. All parishes are represented by extremely poor to poor resilience, with Washington, St. Tammany, and Ascension parishes displaying the poorest resilience scores (<1.0). St. John the Baptist and St. James parishes display the poorest HWBI scores (<50), while Washington, Tangipahoa, East Baton Rouge, and Iberville parishes show the poorest DISC scores (<50).

Based on the scores provided in Table 1 and Figure 4, four parishes were selected for further review as requiring significant resilience capacity building—East Baton Rouge, St. Helena, St. John the Baptist, and Washington parishes. All four parishes have very poor resilience, poor parish-level well-being, high levels of environmental pollutant exposure, and high levels of climate and socioeconomic inequities.

### 3.1. The Selected Parishes

East Baton Rouge Parish is the most populous parish among those examined. In 2020, its population was 456,781, with 161,536 households and 95,243 families. Baton Rouge is the parish seat as well as Louisiana’s state capital. East Baton Rouge Parish is part of the Greater Baton Rouge area. The parish comprises 470 sq mi (1200 km^2^), of which water represents 15 sq mi (39 km^2^) (3.2%). Of the population, 8.3% speak a language other than English at home, and 5.7% are foreign-born.

St. Helena Parish is commonly called one of the Florida parishes (Tangipahoa, Livingston, East Baton Rouge, St. Helena, and East Feliciana parishes). Its population in 2021 was 10,912. The geographic landscape of St. Helena Parish is dominated by piney woods and rolling hills in the northern portion and flat woods and coastal plains in the southern portion. The elevation is approximately 220 feet. This quiet parish is rural, without railways, traffic lights, or significant waterways. A portion of St. Helena’s economy is based on the timber industry, beef cattle, egg production, dairying, and truck farming.

St. John the Baptist Parish, at the time of the 2020 census, had a population of 42,477. Edgard is the parish seat and an unincorporated area. LaPlace is the largest city and is also unincorporated. The parish is presently a part of the New Orleans–Metairie metropolitan statistical area. The oil industry is the primary employer in the region and contributes to several environmental effects. The environmental cancer risk from air pollution in this parish is among the highest of any census tract in the United States [29,30].

Washington Parish is one of the Florida parishes in the interior of southeastern Louisiana. As of the 2020 census, the population was 45,463. Franklinton is its parish seat, and Bogalusa is its largest city. The parish was founded in 1819. According to the U.S. Census Bureau, the parish area is 676 square miles (1750 km^2^). Of this area, 670 square miles (1700 km^2^) is land, and 6.4 square miles (17 km^2^) (0.9%) is water.

### 3.2. Deconstruction of Resilience Indicators

**Figure 5.**
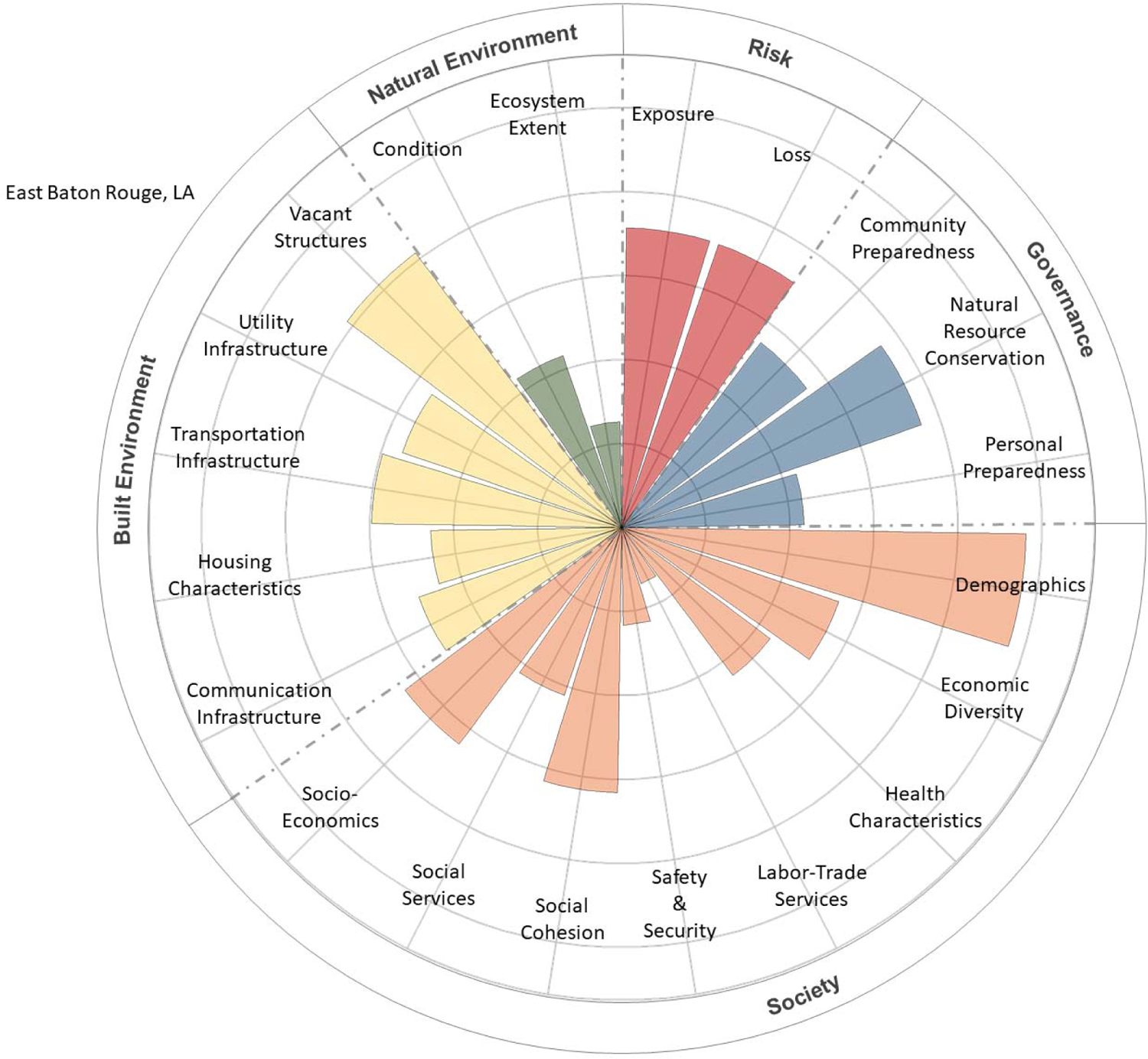
Polar plot of the contributions of CRSI indicators to the domain scores for East Baton Rouge Parish, LA.

**Figure 6.**
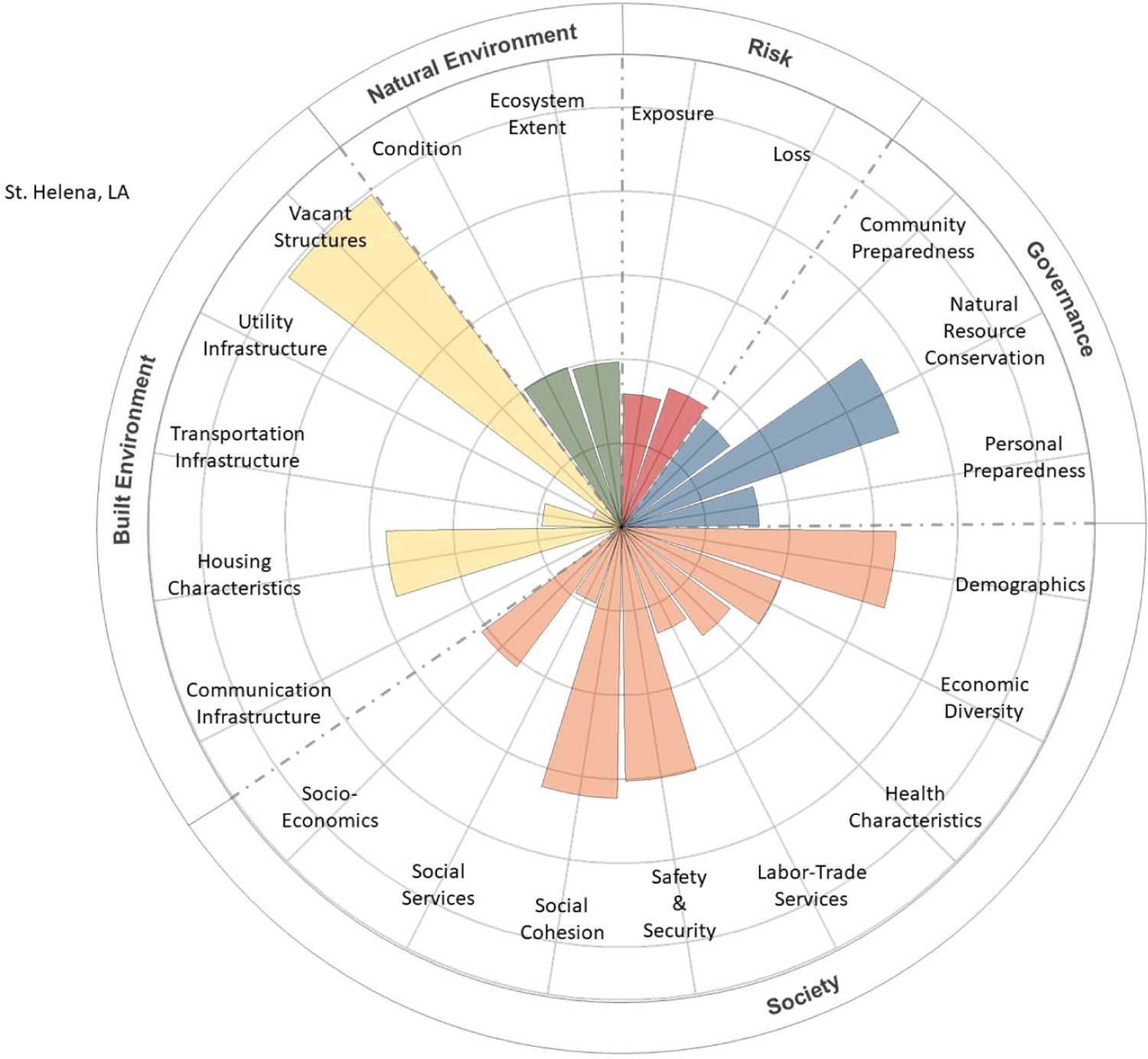
Polar plot of the contributions of CRSI indicators to the domain scores for St. Helena Parish, LA.

**Figure 7.**
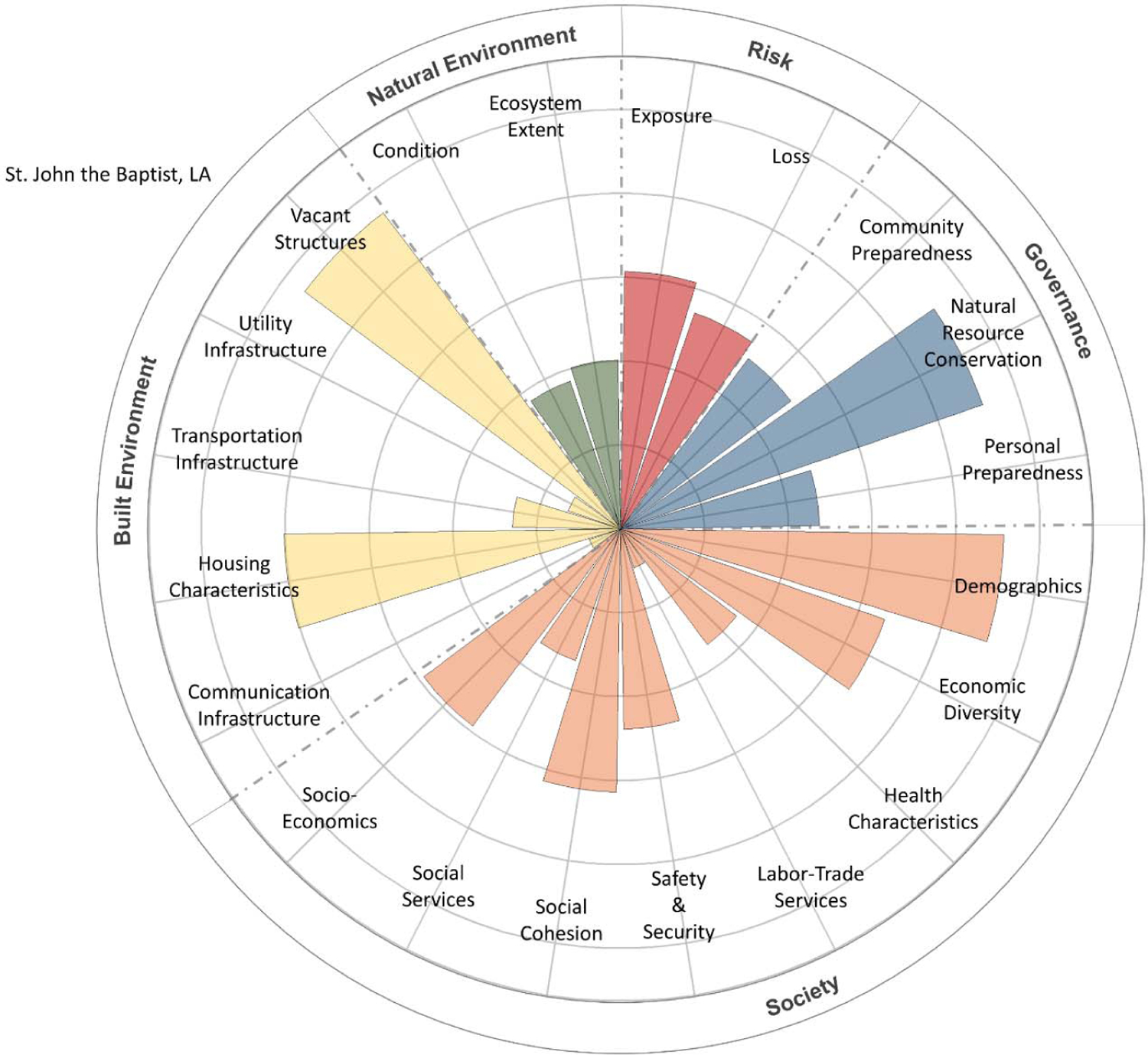
Polar plot of the contributions of CRSI indicators to the domain scores for St. John the Baptist Parish, LA.

**Figure 8.**
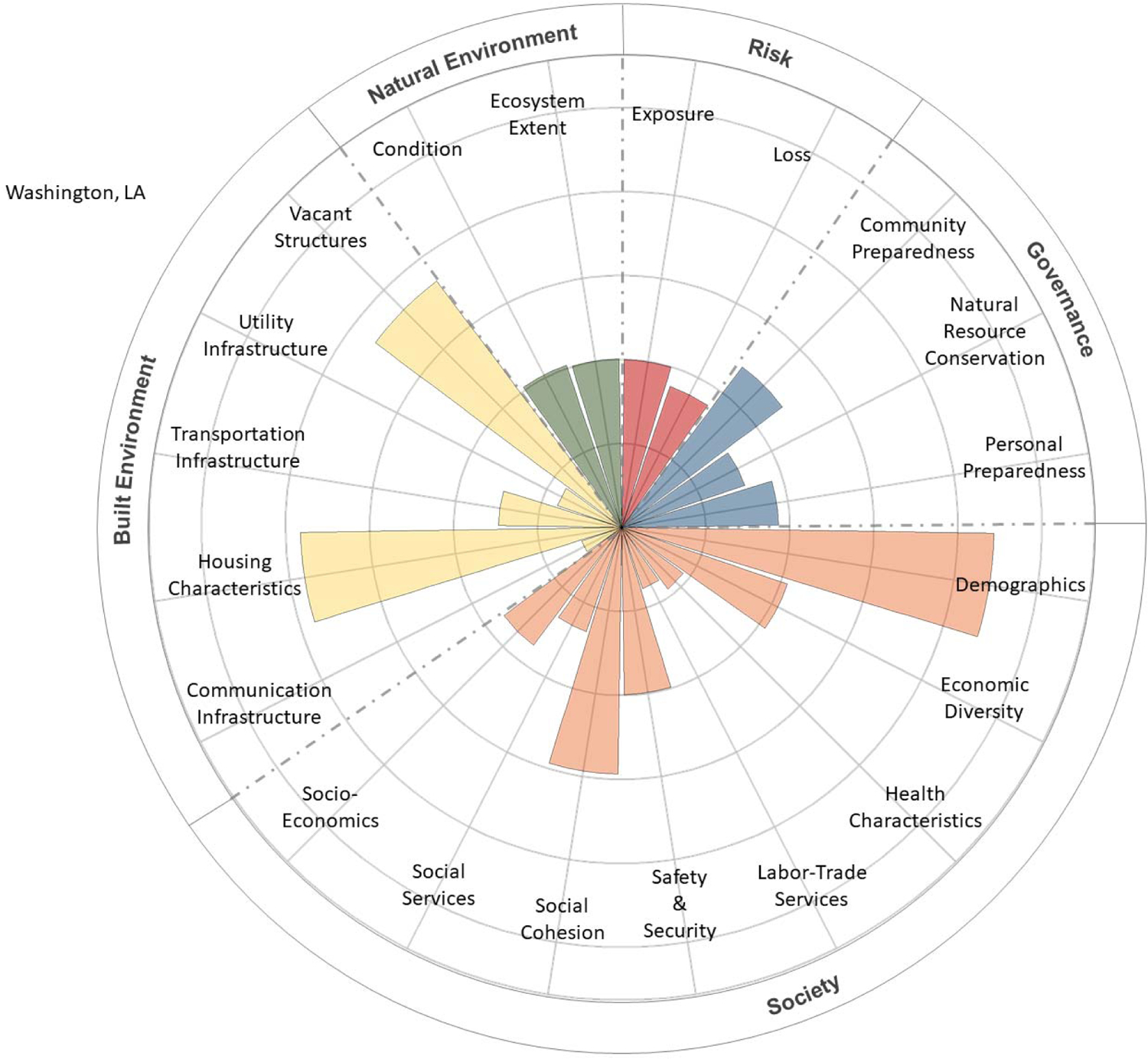
Polar plot of the contributions of CRSI indicators to the domain scores for Washington Parish, LA.

The contributions of CRSI indicators to the five CRSI domains are shown in Figures 5–8 for the selected four parishes. Significant contributions from several common indicator areas (both high and low) were shown to reduce the parish’s CRSI overall index values significantly in all four parishes. In East Baton Rouge Parish (Figure 5), very high contributions from some demographic and vacant structure indicators reduce the parish’s resilience score. Low labor–trade services, safety and security, social services, ecosystem extent, housing characteristics, and built-environment infrastructure (i.e., communications, utilities, and transportation) indicate that scores further reduce East Baton Rouge’s resilience. In St. Helena Parish (Figure 6), extremely high vacant structure indicator scores and extremely low built-environment infrastructure indicator scores drive the parish’s low CRSI score. These attributes, coupled with poor social services, labor–trade services, health characteristics, economic diversity, and personal/community preparedness indicators, result in poor resilience for the parish. High numbers of vacant structures and poor housing characteristics coupled with very poor built-environment infrastructure and low levels of social services, labor–trade services, and health characteristics contribute to poor resilience for St. John the Baptist Parish (Figure 7). Extremely high numbers of vacant structures, skewed demographics, poor housing characteristics coupled with poor built-environment infrastructure levels, poor social services, poor labor–trade services, and poor health characteristics contribute to Washington Parish’s low CRSI score (Figure 8).

Further metric deconstruction of indicator scores associated with these four parishes (Table 2) showed many strongly negative contributions to each parish’s low resilience score (e.g., access to the internet, number of persons/room, incomplete plumbing, special needs transportation, the proportion of population older than 25 years with only 9th-grade education). These negative contributions potentially provide opportunities for resilience capacity building at the parish and community levels. Several of the selected parishes have many poor resilience metrics in common. For example, all generally have low levels of communications infrastructure, while some also show lower levels of utilities and transportation infrastructure. Large numbers of vacant structures and some poor housing characteristics characterize all four parishes. All show a general lack of reinvestment of Small Business Administration funding in mitigation activities. Generally, few households participate in the National Flood Insurance Program despite the potential risks of high flooding. The parishes show mixed conditions associated with protecting natural lands and biodiversity and losses of agricultural and natural lands to impervious surfaces. However, all four parishes show human life and property losses due to natural hazard events. All four parishes display lower levels of ambulance services and, generally, all labor–trade services except steel fabrication in St. Helena Parish. All parishes show lower levels in several social services, especially blood availability, food services for the needy, special needs transportation services, and the number of social advocacy groups. All four parishes appear to have some demographic and health characteristics in common. Many have high proportions of the population without high school diplomas, and many appear to have bimodal distributions of their population with large numbers of elderly and very young people. Large numbers of people with asthma and diabetes populate these parishes. Similarly, many special needs persons who cannot quickly evacuate during natural hazard events populate these parishes. Half the parishes have high levels of deep poverty, while one, namely St. John the Baptist Parish, shows high economic inequity.

The evaluation of these community resilience and well-being indicators and environmental, climate, and social justice characteristics, coupled with practical political considerations determined by CRPC (e.g., the closeness of the next mayoral election, desire of community officials to work with CRPC in the past), resulted in St. Helena Parish being selected as the parish for further metric assessment and community engagement.

## 4. Discussion

The CSRI and CEJST results for the four candidate parishes within CRPC jurisdiction showed a distinct tendency toward poor resilience equating with underdeveloped infrastructures, poor housing characteristics, poor preparedness, skewed demographics, and poor economic conditions compared to the remaining counties comprising the United States. Internet access and enhanced public libraries are well established as positive contributors to community resilience [31–33]. Internet access in the four candidate parishes is among the bottom 1% of counties/parishes in the United States and potentially contributes to the parishes’ poor resilience. Similarly, the role of the transportation network is vital for community resilience [34]. The candidate parishes are in the bottom 7% of counties/parishes with ready highway access. Energy-producing facilities, wastewater treatment facilities, and redundancies built into both infrastructures contribute positively to community resilience [35,36]. Except for East Baton Rouge Parish, which is in the top 1% of U.S. counties/parishes for wastewater facility infrastructure and the top 50% of U.S. counties/parishes for power plant infrastructure, the remaining candidate parishes rank in the bottom 28% of counties/parishes for power plant, wastewater, and drinking water infrastructures. St. Helena Parish is among the bottom 1% of U.S. counties/parishes for power plant and wastewater infrastructure facilities.

Poor housing conditions and abundant vacant structures clearly reduce a community’s resilience to climatic hazards [36–38]. Three of the four candidate parishes are ranked in the top 4% of counties with the most vacant structures. Only Washington Parish showed fewer vacant business structures, being in the country’s top 12% of counties/parishes. All four candidate parishes are among the worst (top 1%) counties/parishes for overcrowding of homes (>1.5 persons per room) and incomplete plumbing.

Community and personal preparedness similarly play an essential role in community resilience [39]. All four candidate parishes are among the bottom 1% of U.S. counties/parishes in reinvestment of Small Business Administration mitigation funds. Although they face high flooding risks, all the candidate parishes are in the bottom 20% of U.S. counties/parishes participating in the National Flood Insurance Program. Generally, about 50–75% of these non-participating U.S. counties and parishes are in low-flood-risk areas.

Demographic attributes like health, social cohesion, and distribution play a role in resilience at the community level [40–42]. The diversity of labor–trade services can play a positive role in recovery from climatic events [23]. Generally, the four candidate parishes are among the bottom counties/parishes in the U.S. concerning the number of concrete construction, framing construction, water and sewer construction, masonry and roofing construction, and steel fabrication companies located in the parish. The number and availability of these types of companies are often critical for parish recovery after an event. Low educational attainment levels in the candidate parishes are also apparent, generally in the lower 10% of U.S. counties/parishes. Similarly, high proportions (>60%) of the populations of St. Helena and Washington parishes being below the poverty level would also contribute to reduced resilience.

### Next Steps

The use of existing information to assess the resilience capacity of communities is relatively common [19,21,22], but this information is only sometimes confirmed with the communities assessed. The use of local knowledge [43–46] outside the United States (except for tribal traditional ecological knowledge [47,48]) and community engagement [49–52] are two tools that can be used to confirm available information, initiate discussions on the development of strategies to build local resilience capacity, and institute changes in local policies to improve resilience.

Community engagement is an important next step for creating and enabling community resilience. It generates a strong sense of inclusion and belonging [10]. There are four common knowledge types for developing community resilience [18]. Scientific knowledge draws from professional and academic arenas and is generally considered the most accurate. Political or bureaucratic knowledge describes the long-term tenets of the community based on policies, laws, and formal practices. Local knowledge represents a community’s institutional memory and often determines how relevant community members view threats. Usually, local knowledge does not have a scientific basis, but it does contribute a sense of local understanding. Finally, indigenous knowledge comes from the practices and beliefs embedded in First Nations peoples’ culture and practices.

Professional practice and literature support that community engagement is usually best done through a bottom-up approach [53,54]. This bottom-up approach integrates lived community member experiences into resilience approaches. Engagement at the grassroots level helps improve individual resilience by enhancing social capital while enabling the development of social networks [55].

The discussion process is fundamental to community engagement [56]. These discussions must be inclusive, describe the definition of resilience, and define how resilience would work in the community. The correct type of engagement can foster enhanced human capital that meets community requirements to improve the transition between short- and long-term recovery [57]. Community engagement approaches must be inclusive and genuine, taking a bottom-up approach to meet top-down information to ensure proper understanding. The approach described in the CRPC project uses existing information to provide a starting point for community engagement. We intend to interact with the St. Helena community (government entities and the overall community) to assess how much current information matches local knowledge, adjust our understanding if required, and develop strategic approaches to building community resilience capacity.

The initially intended community engagement will be focused on several questions designed to elicit local knowledge and provide confirmatory information concerning the information described here. Examples of these questions for governmental, NGO, and citizen entities would include the following:

1. Do you believe St. Helena Parish and its communities would benefit from resilience capacity building related to natural hazard events?
2. What are the parish’s greatest strengths and most significant shortcomings regarding these natural hazard events?
3. Can the communications infrastructure for the parish be improved? If so, how?
4. Do you believe there are a large number of vacant structures in the parish? What types? Would they contribute to poor parish-level resilience?
5. Would you consider the parish at high risk for loss of human life and high levels of property loss due to natural hazard events?
6. Could labor–trade services and social services for the parish be improved? How?
7. Do you believe the parish has serious levels of health issues like asthma and diabetes?
8. Do you believe the parish has an unacceptable proportion of families experiencing deep poverty? Does the parish display economic, environmental, climatic, or social inequities?

These questions can provide a basis for engagement to determine local knowledge’s congruence and the information types provided here. Suppose an agreement can be reached between the community members (government, NGOs, and citizens) and the project. In that case, the groups can jointly develop and assess strategies to improve the resilience capacity of St. Helena Parish.

## 5. Conclusions

The information provided here represents the first step in a five-step approach to developing and enhancing resilience to natural hazard events in southeastern Louisiana. The assessment identified St. Helena Parish as an ideal candidate for resilience capacity building. This initial phase of the resilience capacity building project, namely selecting target communities/parishes with local (CRPC) assistance, is described in detail. The examination identified multiple shortcomings that could be improved to enhance St. Helena Parish’s resilience to natural hazards, including internet access, infrastructure improvements, reducing the number of vacant structures, housing improvements, community preparedness, and social services enhancements. The project’s next phase—confirmation of these identified shortcomings and information through community engagement—has been outlined and will be initiated in 2024. Upon this confirmation (or lack thereof) based on local knowledge, the community and project members will develop strategies and intervention approaches to build, enhance, and strengthen the overall resilience capacity of the parish. These strategies will be pursued and implemented if feasible. Upon implementation, the success of the resilience capacity enhancements will be monitored, and the transferability of the approach to other parishes in Louisiana and counties in the United States will be evaluated.

The development of resilience capacity in St. Helena Parish will contribute to the long-term sustainability of the parish. Correcting a number of shortcomings in resilience that have been identified in this manuscript will improve the ability of the parish and its residents to prepare for and respond to climatic events, particularly flooding and hurricanes. Initial conversations with St. Helena Parish local government officials confirm a number of the identified resilience issues as problems in the parish (e.g., the lack of broadband access). This lack has historically been problematic for the communication of directives for hurricane egress and has affected the long- and short-term sustainability of the parish. The development of strategies and policies to address a number of the identified resilience shortcomings for the parish will enhance not only its resilience to events but also its sustainability.

## Figures and Tables

**Table 1.** Results of the Cumulative Resilience Screening Index (CRSI), the Human Well-Being Index (HWBI), the Decision Integration for Stronger Communities Index (DISC), the Environmental Justice Screening Tool (EJScreen), and the Climate and Economic Justice Screening Tool (CEJST) for targeted parishes in southeastern Louisiana in the Capital Region Planning Commission (CRPC) and Louisiana Watershed District #7 (LW #7).

Region	Parish	CRSI	HWBI	DISC	EJScreen	CEJST
					Mean Score	% Census Tracts Listed as True
CRPC and LW #7	Ascension	0.889	54.2	55.1	14.3	21.43
	East Feliciana	1.270	47.0	50.1	61.2	60.00
	**East Baton Rouge**	**1.325**	**47.8**	**50.0**	**73.9**	**46.73**
	Iberville	1.600	46.8	49.7	73.3	71.43
	Livingston	1.392	54.0	55.5	13.3	11.76
	**St. Helena**	**1.305**	**47.9**	**50.6**	**60.7**	**50.00** *
	Tangipahoa	2.005	48.2	49.6	61.5	65.00
	**Washington**	**0.882**	**48.3**	**49.2**	**66.9**	**90.91**
	West Feliciana	2.169	52.8	55.3	56.1	33.33
LW #7 only	St. Charles	1.642	55.0	55.2	28.0	23.08
	St. James	1.549	50.7	52.4	59.8	57.14
	**St. John the Baptist**	**1.591**	**51.6**	**54.3**	**77.3**	**63.64**
	St. Tammany	0.968	53.1	53.5	15.4	18.60
CRPC Only	Pointe Coupee	2.348	53.0	54.5	57.7	50.00
	West Baton Rouge	1.721	53.1	54.2	52.0	40.00
Louisiana	All Parishes Combined	2.501	49.0	52.0		
United States	All Counties Combined	3.060	54.1	54.7		
Mean for CRPC Parishes		1.537	50.3	52.2	53.7	49.15

(* =One of two census tracts in St. Helena Parish listed as True.)

(Bold demarks the four parishes selected for further review.) All scores run, theoretically, from 0 to 100, although most CRSI scores are between 0 and 10.

**Table 2.** Select deconstructed metrics important to four CRPC/LW#7 parishes with very low resilience scores. Values shown in terms of percentage of counties in the United States with similar metric scores. For example, East Baton Rouge, St. Helena, St. John the Baptist, and Washington parishes are all in the lowest 1% of counties/parishes in the U.S. for Access to Internet.

Indicator	Metric	East Baton Rouge	St. Helena	St. John The Baptist	Washington
Communications Infrastructure	Access to Internet	Bottom 1%	Bottom 1%	Bottom 1%	Bottom 1%
	Number of Mobile Broadcast Towers	Bottom 11%	Bottom 1%	Bottom 1%	Bottom 1%
	Number of Paging Transmission Towers	Bottom 50%	Bottom 1%	Bottom 8%	Bottom 1%
	Number of Radio Broadcast Towers	Bottom 39%	Bottom 3%	Bottom 2%	Bottom 11%
	Number of TV Station Transmitters	Top 88%	Bottom 1%	Bottom 1%	Bottom 1%
Transportation Infrastructure	Access to Highways	Bottom 5%	Bottom 7%	Bottom 5%	Bottom 1%
Utilities Infrastructure	Power Plant Facilities	Top 50%	Bottom 1%	Bottom 1%	Bottom 16%
	Wastewater Treatment Facilities	Top 1%	Bottom 1%	Bottom 28%	Bottom 8%
	Drinking Water Facilities	Bottom 16%	Bottom 19%	Bottom 13%	Bottom 24%
Vacant Structures	Business	Top 1%	Top 1%	Top 4%	Top 12%
	Residential	Top 3%	Top 1%	Top 1%	Top 4%
	Other	Top 1%	Top 1%	Top 1%	Top 1%
Housing Characteristics	Over 1.5 persons/room	Top 1%	Top 1%	Top 1%	Top 1%
	Incomplete Plumbing	Top 1%	Top 1%	Top 1%	Top 2%
	Mobile Homes	Top 1%	Bottom 27%	Top 7%	Top 11%
Community Preparedness	Re-investment of SBA* in mitigation	Bottom 1%	Bottom 1%	Bottom 1%	Bottom 1%
Personal Preparedness	Participation in NFIP**	Bottom 7%	Bottom 20%	Bottom 17%	Botto 17%
Natural Resource Conservation	Protected Lands	Bottom 1%	Top 31%	Top 10%	Bottom 31%
	Loss to Impervious Surface	Top 99%	Top 95%	Top 26%	Bottom 5%
	Protecting Biodiversity	Top 60%	Top 57%	Top 72%	Bottom 31%
Indicator	Metric	East Baton Rouge	St. Helena	St. John The Baptist	Washington
Risk	Loss of Human Life And Property	Top 14%	Top 1%	Top 1%	Top 1%
Safety and Security	Ambulance Services	Bottom 1%	Bottom 1%	Bottom 19%	Bottom 33%
Labor–Trade Services	Concrete Construction	Bottom 7%	Bottom 1%	Bottom 18%	Bottom 7%
	Framing Construction	Bottom 7%	Bottom 1%	Bottom 1%	Bottom 6%
	Water and Sewer Construction	Bottom 7%	Bottom 26%	Bottom 6%	Bottom 5%
	Masonry, Power Generation, Roofing	Bottom 10%	Bottom 1%	Bottom 6%	Bottom 15%
	Steel Fabrication	Bottom 17%	Top 4%	Bottom 1%	Bottom 41%
Social Services	Surgical Services	Bottom 29%	Bottom 1%	Bottom 27%	Bottom 25%
	Blood Availability	Bottom 7%	Bottom 1%	Bottom 1%	Bottom 1%
	Food Services for the Needy	Bottom 7%	Bottom 1%	Bottom 1%	Bottom 23%
	Number of Insurance Claims Establishments	Bottom 47%	Bottom 1%	Top 37%	Bottom 26%
	Mental Health Establishments	Bottom 36%	Bottom 1%	Bottom 12%	Bottom 1%
	Inpatient Nursing and Rehab Services	Bottom 16%	Bottom 1%	Bottom 6%	Bottom 17%
	Social Advocacy Groups	Bottom 15%	Bottom 1%	Bottom 1%	Bottom 17%
	Special Needs Transportation	Bottom 13%	Bottom 1%	Bottom 1%	Bottom 1%
	Number of Mental Health Professionals	Bottom 36%	Bottom 1%	Bottom 12%	Bottom 1%
	Basic School Facilities	Bottom 33%	Top 53%	Bottom 36%	Bottom 15%
Demographics	Population > 25 yrs and Education < 9th Grade	Top 1%	Top 31%	Top 5%	Top 9%
	Population < 14 yrs with Limited English	Top 2%	Top 33%	Top 8%	Top 4%
	Proportion of Population Without HS Diploma	Top 1%	Top 41%	Top 10%	Top 11%
	Proportion of Population < 5 yrs	Top 1%	Top 33%	Top 9%	Top 8%
Indicator	Metric	East Baton Rouge	St. Helena	St. John The Baptist	Washington
Demographics	Population > 65 yrs and Living Alone	Top 25%	Top 60%	Top 59%	Top 37%
Health Characteristics	Incidence of Adult Asthma	Top 8%	Top 8%	Top 8%	Top 8%
	Incidence of Child Asthma	Top 9%	Top 9%	Top 9%	Top 9%
	Incidence of Diabetes	Top 11%	Top 15%	Top 12%	Top 15%
	Special Needs with Limited Ability to Evacuate	Top 13%	Top 23%	Top 15%	Top 23%
Socioeconomics	Population Below Poverty Level	Top 39%	Top 62%	Top 36%	Top 60%
Economic Diversity	Income Disparity (GINI Score)	Top 64%	Top 61%	Top 39%	Top 67%

* = Small Business Administration;

** = National Flood Insurance Program.

## Data Availability

The data presented in this study are available on request from the corresponding author.
